# So much data, so little time: Using sequential data analysis to monitor behavioral changes

**DOI:** 10.1016/j.mex.2016.10.004

**Published:** 2016-10-22

**Authors:** Tywanquila Walker

**Affiliations:** Department of Psychology, Cornell University, United States, United States

**Keywords:** Sequential data analysis, Sequential data analysis, Family interactions, Infant behavior, Caregiver responsiveness

## Abstract

Twenty-three infants (*M* = 13.7 months, *SD* = 3.73) and their primary caregivers were observed and video-taped in three 20-min play sessions. Over the course of a month, changes in infant behaviors and caregiver responsiveness to those behaviors were monitored. Repeated-measures ANOVAs indicated that caregiver responsiveness to infant object-related and dyadic behaviors significantly increased over the course of the sessions. However, the ANOVAs did not specify exactly which caregiver behaviors changed. Sequential data analysis revealed that caregivers specifically increased their use of dyadic vocal behaviors in response to all infant behaviors. This study reveals that although ANOVAs are useful for providing information about macro, overall changes in caregiver behavior, sequential data analysis is a useful tool for evaluating micro, moment-to-moment changes in behavior. With sequential analysis, specific behavioral patterns can be examined and, if necessary, steps can be taken to modify and monitor those behaviors over time.

• Sequential data analysis was used to monitor changes in caregiver behavior.
• Non-culture-specific behavioral codes and techniques were used to quantify caregiver responsiveness to infant object-related and dyadic behaviors.
• When compared to ANOVA, sequential data analysis is more useful for assessing micro-level behavioral changes in infant-caregiver interactions.

## Graphical abstract

**Figure:**
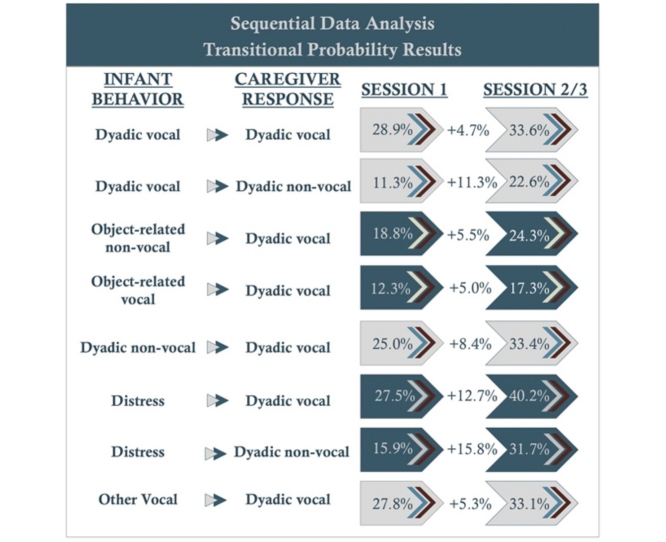

## Introduction and rationale

Studies with at-risk families have consistently shown that parental responsiveness affects children’s development. In terms of familial interactions and communicative behaviors, compared to children who are raised in families with higher socio-economic statuses, children in low-income households have fewer words spoken directly to them in their first year of life [1], [2]. In an observational, longitudinal study of parent-child verbal interactions, Hart and Risley [1] showed that children in professional families heard an average of 2153 words per hour, children in working class families heard an average of 1251 words per hour, and children in welfare families heard an average of 616 words per hour. In a year, it was estimated that children in professional, working class, and welfare families heard, on average, 11 million, 6 million, and 3 million words, respectively. By age three, the observed cumulative vocabulary for children in professional families was about 1100 words, 750 words for children in working families, and 500 words for children in welfare families.

By the time these children reach first grade, it is already apparent that they are behind their more privileged counterparts in terms of academic achievement (see e.g., [3], [4], [5], [6]). Furthermore, having a low vocabulary may lead to low performance in school, low test scores, and high dropout rates. These results provide clear evidence that the way parents communicate with their children affects subsequent cognitive and linguistic development.

Similarly, longitudinal and intervention studies indicate that caregiver responsiveness significantly impacts children’s vocabulary development and rate of learning (e.g., [7], [1], [8], [9], [2]). The results of these studies provide evidence that caregiver responsiveness is a key component of infant cognitive development. Yet what is the pattern of these infant and caregiver behaviors? Specifically, which infant behaviors prompt responsiveness and which types of responsive behaviors are caregivers exhibiting?

In order to assess caregiver-specific behavior and responsiveness, we must examine the micro-level, moment-by-moment interactions of infants and caregivers. Sequential data analysis (see e.g., [10], [11], [12], [13], [14]) is a statistical method that allows us to examine these patterns of behavior over time. Using sequential data analysis, we can determine which behavioral sequences occur at rates that are significantly greater than chance. Additionally, because it is difficult to code every infant or caregiver behavior during a live observation, video recordings can be used to observe the moment-to-moment interactions that occur between caregiver and infant. Specifically, the video recordings can be used to keep track of infant vocalizations and behaviors as well as caregiver responses.

The current research quantifies even the smallest changes in caregiver behavior and identifies the mechanisms that influence the quality of infant-caregiver interactions. Together, video recordings and sequential data analysis capture live, real-time infant-caregiver interactions and allow researchers to assess micro-level changes in caregiving behaviors over time. The statistical techniques presented in this research can be used to help psychologists, educators, and other practitioners identify key parental behavioral responses that impact children’s language and cognitive development.

In sum, research that investigates how caregiver responsiveness impacts the language and cognitive development of children will provide insight into how parents can positively influence their children’s development. Not only will caregiver responsiveness research enhance the development of infants who are at risk for deprivation of communication and/or social interactions, it will also provide parents with information about real-time, responsive behaviors that will benefit their children. Showing parents that their interaction with their infants today has long-term positive effects on their children’s futures may prove to be a strong motivator that encourages parents to enhance infant-caregiver interactions.

## Method

### Participants

Twenty-three infants between the ages of 8 and 24 months (*M* = 13.7 months, *SD* = 3.73; 11 females, 12 males; 8 months [n = 2], 9–16 months [n = 20], 24 months [n = 1]) and their primary caregivers (*M* = 34.6 years, *SD* = 5.77, age range 26–42 years; one primary caregiver was male) participated in this study. Approximately 83% of the participants were from middle- to upper-middle class families. All caregivers had at least a high school diploma. 43.5% had bachelor’s degrees and 39% had master’s degrees or higher. 78.3% of the participants where white, 4.3% black, 4.3% Hispanic, and 13% were either multi-racial or of other ethnicities. Four additional participants were omitted from the study because of equipment failure (1), experimenter error (1), fussiness (1), and failure to return for a second session (1).

Infants were recruited through a letter given to parents at the time of the infant’s birth. Parents interested in participating in studies on infant learning and development were added to a database. To schedule an appointment, parents were sent an email, followed by a telephone call. All infants received a t-shirt, sippy cup, or bib in appreciation for their participation.

### Procedure

Over the course of a month, each dyad was observed and video-taped in three 20-min interactive play sessions that occurred approximately 2.5 weeks apart. At each session, the dyads were given a set of toys to play with and asked to play as they would at home. Session 1 was used as a baseline measure of infant-caregiver interactions and caregiver responsiveness. At the end of Session 1, caregivers received information about caregiving behavior in the form of a 10-min video, which highlighted the importance of talking to infants. “Improving Your Baby’s Language Skills” by talking to your baby was the overall theme of the video. In the video, caregivers were given four tips for talking to their infants: (1) Be Positive and Patient, (2) Act Naturally, (3) Name Objects for Your Baby, and (4) Practice. After the introduction of each tip, caregivers were given strategies for how to interact with their infants. Although a mother and her 18-month-old son modeled tips one and three, the tips were simple and generalizable enough to apply to infants of any age. After watching the video, caregivers were instructed to talk to their infants and practice the caregiving behaviors that were demonstrated in the video.

The purpose of the video was to encourage caregivers to interact more with their infants, and thus measure behavioral changes from baseline at subsequent sessions. Sessions 1, 2, and 3 were compared to test the effect of the manipulation and measure behavioral changes over the course of the month. Caregivers did not receive additional caregiving information at Sessions 2 or 3. Approximately 61% of families participated in all three sessions (*n* = 14).

### Behavioral measures and coding

The play sessions were coded for infant behaviors and the corresponding behaviors of their caregivers. Caregiver responsiveness was coded using a system based on Gros-Louis, West, Goldstein, and King [15], Bornstein et al. [16], and Vollmer [17]. The following behaviors were observed and quantified for both the caregiver and the infant: object-related non-vocal, object-related vocal, dyadic non-vocal, dyadic vocal, and other vocal. Additionally, distress vocalizations were coded for infants and other object-related non-vocal for caregivers (Table 1).

As this study focuses on caregiver responsiveness, the coding of all behaviors was infant driven. If a caregiver responded within five seconds after the infant initiated a behavior, the caregiver was counted as being responsive to that behavior; caregivers could respond in any of the aforementioned ways regardless of the infant’s initial behavior. For example, if an infant made an object-related vocalization and the caregiver responded (within five seconds) with a dyadic non-vocalization, the caregiver’s behavior counted as responsive. On the other hand, if the caregiver failed to respond, or responded in greater than five seconds, the behavior was not counted as responsive. Any caregiver behavior that was categorized as “other”, even if it occurred within five seconds of an infant behavior, was not counted as responsive (i.e., the caregiver was not focused on the infant and thus was not responsive to the infant).

Caregiver responsiveness to a behavior was calculated as the number of times a caregiver responded to that behavior divided by the number of times the infant exhibited the behavior. For example, if an infant exhibited 100 infant objected-related non-vocalizations and the caregiver was responsive 52 times, the caregiver’s level of responsiveness to infant object-related non-vocalizations was 0.52 (i.e., 52/100 = 0.52). Thus, caregiver responsiveness for each behavior was calculated as a proportion.

#### Intercoder reliability

All videos were coded by two coders. Prior to coding for this study, each coder completed approximately six hours of video-coder training; the author trained both coders. Training consisted of coding three 20-min practice videos, which were recorded for another infant-caregiver study. Practice videos were chosen such that the caregivers varied in level of responsiveness and types of caregiver behaviors. Throughout training (i.e., before, during, and after coding sessions), coders and the author discussed the behavior categories and how each infant or caregiver behavior should be coded. After each practice video was coded, the author compiled a spreadsheet and showed the coders their reliability measures. Reliability was calculated using percent agreement. Discrepancies were discussed and, if necessary, the practice video was recoded. When intercoder reliability reached greater than 90% for the third practice video, coding began for the current study.

On average, it took coders 1 to 1.5 hr to code a 20-min video. To check intercoder reliability, four videos were randomly selected for coding. The first 10 min of each video was coded, which resulted in 40 min of recordings for reliability checks. Percent agreement ranged from 78% to 98%. Intercoder reliability was also measured by computing intraclass correlation coefficients (ICC). ICCs ranged from 0.70 to 0.98. Coder 1 coded 36% of the videos for this study and Coder 2 coded 64% of the videos.

### ANOVA vs. sequential data analysis

#### ANOVA

The frequency of each infant behavior and caregiver level of responsiveness to each behavior was analyzed. Level of responsiveness to infant behavior was calculated as a proportion and changes in caregivers’ level of responsiveness at Sessions 1, 2, and 3 were examined. Repeated-measures ANOVAs, using a Bonferonni correction, indicated that caregiver responsiveness to infant object-related non-vocal, object-related vocal, dyadic non-vocal, and dyadic vocal behaviors did not significantly differ from Session 2 to Session 3, nor was there a difference in overall responsiveness between these two sessions (all *p* > 0.05). Therefore, the data for these two sessions was combined into a category that is referred to as Session 2/3.

**Fig. 1:**
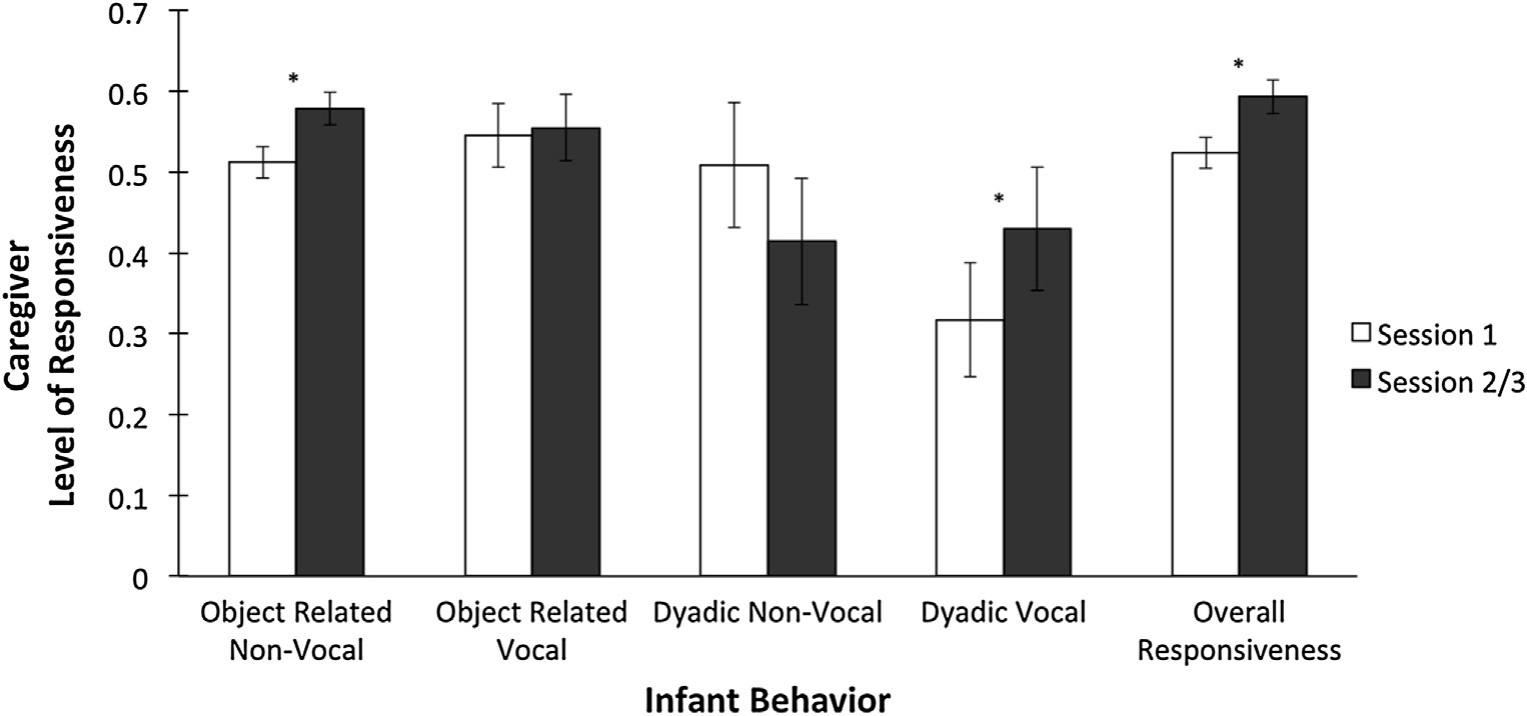
Caregiver level of responsiveness to infant behaviors from Session 1 to Session 2/3. Significant differences are denoted by an asterisk (*). Error bars represent standard errors.

Repeated measures ANOVAs determined that caregiver responsiveness to object-related non-vocal (*F*(1, 22) = 8.30, *p* = 0.009, η^2^ = 0.27) and dyadic vocal (*F*(1, 22) = 5.10, *p* = 0.034, η^2^ = 0.19) behaviors significantly increased from Session 1 to Session 2/3. Overall responsiveness (*F*(1, 22) = 8.34, *p* = 0.009, η^2^ = 0.27) also increased significantly. However, responsiveness to object-related vocal (*F*(1, 22) = 0.03, *p* = 0.87, η^2^ = 0.001) and dyadic non-vocal (*F*(1, 22) = 2.39, *p* = 0.14, η^2^ = 0.10) behaviors was not significantly different, although there was a decreasing trend for dyadic non-vocal behaviors (Fig. 1).

#### Sequential data analysis

Lag 1 sequential data analyses of repeating and non-repeating consecutive behaviors [18] were conducted to assess changes in caregiver responsiveness from Session 1 to Session 2/3. For both types of sequential analyses, we looked at all infant and caregiver behaviors and analyzed the transitional probability that one behavior would follow another. Thus, sequential analyses were used to identify recurring behavioral patterns. Both infant- and caregiver-initiated behaviors were examined.

Sequential analyses allowed us to more closely examine the frequency and transitional probability from infant behavior to caregiver behavior and vice versa. The analyses provided a bi-directional viewpoint and allowed us to determine which infant behaviors caregivers were most likely to respond to as well as which caregiver behaviors infants were likely to respond to. In particular, we looked at behavioral patterns and examined whether certain behaviors were more likely to follow others.

The major distinction between the two types of sequential analyses is that repeating consecutive behavior analyses examine the transition from one type of behavior to another, regardless of whether the previous and following behaviors are the same. In contrast, non-repeating analyses focus on the transition from one behavior to a new or different behavior. Repeating analyses can be used to identify recurring behaviors, whereas non-repeating analyses can be used to identify changes in behaviors or states. Both methods are useful for identifying micro-level changes in infant-caregiver interactions.

For repeating consecutive behavior analyses, all behaviors were analyzed. For example, a behavioral sequence of infant dyadic vocal (idv), infant dyadic vocal (idv), caregiver dyadic vocal (cdv), infant object-related non-vocal (ion) would result in an idv to idv frequency of 1 and a transitional probability of 0.50. In contrast, in a non-repeating consecutive behavior analysis, consecutive repeating behaviors were excluded. Thus, a behavioral sequence of idv, idv, cdv, ion would be collapsed into an idv, cdv, ion sequence, resulting in an idv to idv frequency of 0 and a transitional probability of 0. Sequential data analysis comparisons were performed using O’Connor’s [19] SEQGROUPS SPSS syntax program.

**Fig. 2:**
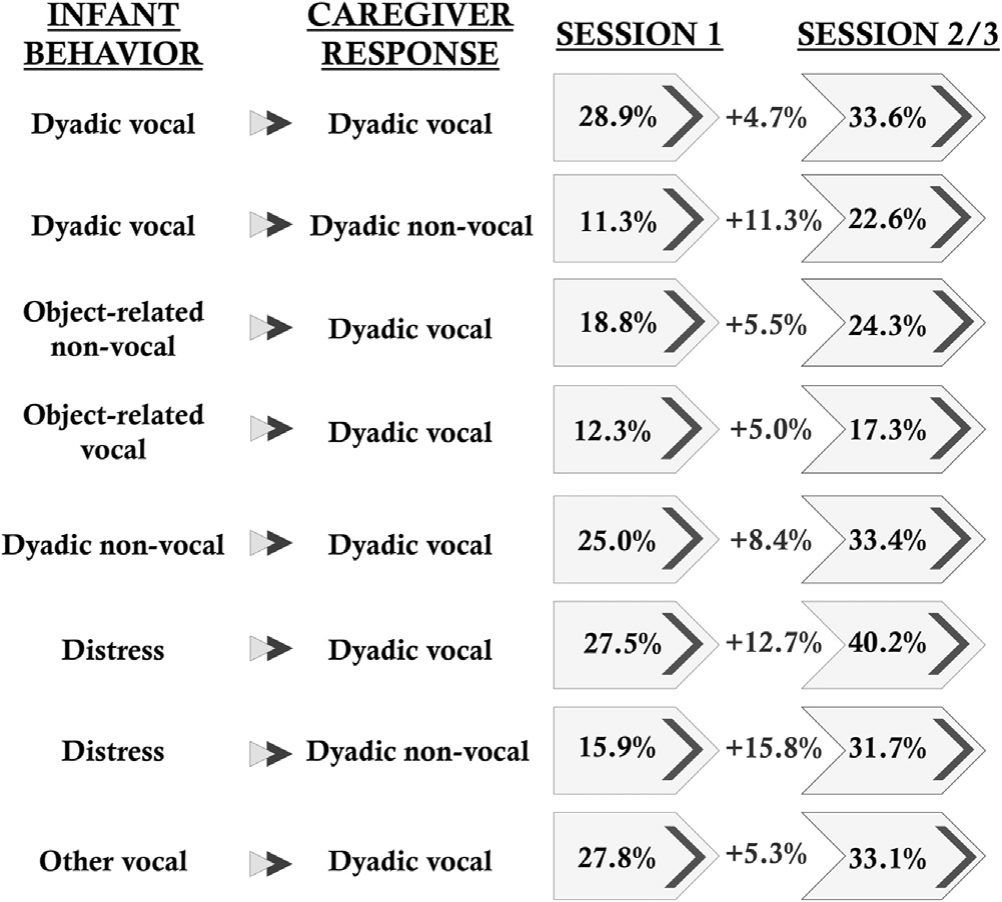
Repeating consecutive behavior transitional probabilities. Percent increase of caregivers’ dyadic vocal responses to infant behaviors from Session 1 to Session 2/3. All percent increases are statistically significant, *p* < 0.001.

Repeating consecutive behavior comparisons, based on likelihood ratio chi-square tests, revealed that there were significant differences in the behavioral patterns of caregivers from Session 1 to Session 2/3 (Likelihood Ratio Chi-Square *χ^2^* = 866.72, *df* = 156, *p* < 0.001). Most notably, caregivers’ dyadic vocal responsiveness to all infant behaviors increased (Table 2, Table 3, and Fig. 2). A comparison of transitional probabilities show that caregiver dyadic vocal responsiveness to infant dyadic vocal behaviors increased by 4.7% (adjusted residual *z* = 9.114, *p* < 0.001), object-related non-vocal behaviors by 5.5% (*z* = 18.02, *p* < 0.001), object-related vocal by 5% (*z* = −6.167, *p* < 0.001), dyadic non-vocal by 8.4% (*z* = 9.967, *p* < 0.001), infant distress/cries by 12.7% (*z* = 5.236, *p* < 0.001), and other infant vocal behaviors by 5.3% (*z* = 10.651, *p* < 0.001) (Fig. 2).

Caregiver and infant behavior responsiveness patterns were also examined using non-repeating consecutive behavior analyses. As was noted with repeating analyses, caregivers increased dyadic vocal behavior (Likelihood Ratio Chi-Square *χ^2^* = 843.14, *df* = 156, *p* < 0.001; Table 4 and Table 5). Infant object-related non-vocal responses to other caregiver vocal behaviors increased by 4.2% (*z* = −6.82, *p* < 0.001) and caregiver dyadic vocal responses to infant dyadic non-vocal behavior increased by 9.5% (*z* = 13.45, *p* < 0.001). Both repeating and non-repeating sequential analyses specifically identify changes in caregivers’ dyadic behaviors.

## Additional information—significance and future applications

The present study was designed to test whether sequential data analysis is a viable statistical analysis method for evaluating changes in caregiver responsiveness to infant behaviors. Thus, ANOVAs were used to examine macro, proportional changes in caregiver response levels and sequential data analysis was used to measure micro, transitional probability changes in responsiveness. Both analyses highlight changes in caregivers’ behaviors and indicate that overall caregiver responsiveness significantly increased from Session 1 to Session 2/3. However, the level of detail provided by sequential data analysis allows us to look more closely at the infant-caregiver interaction. Using sequential data analysis, we can track the moment-by-moment interactions of infants and caregivers, which allows us to monitor behavioral changes over time and analyze sequences of behaviors in addition to overall changes in level of responsiveness.

For example, ANOVAs showed increases in caregiver level of responsiveness to infant object-related non-vocal behaviors. Given that caregivers could respond with four possible object-related and dyadic behaviors, it is unclear which caregiver behavior contributed most to the significant increase in responsiveness. However, further examination with sequential data analysis showed that caregivers specifically increased their use of dyadic vocal behaviors. Combined, increased responsiveness to infant object-related non-vocal behavior and increases in caregiver dyadic vocalizations indicate that caregivers were engaging their infants in more dyadic play. Specifically, caregivers were labeling objects and talking to their infants, not simply manipulating objects.

The results of this study are very promising in that they provide evidence that sequential data analysis can be used as a tool for evaluating moment-to-moment behavioral changes in infant-caregiver interactions. Although ANOVAs and other statistical techniques are useful for examining overall, macro-level effects, sequential data analysis provides a micro-level assessment of which caregiver behaviors are most influenced by experimental events (e.g., video manipulation, caregiver interventions) and natural occurrences (e.g., the passage of time, birth of a sibling). These fine-grained, sequential analyses can be used to provide quantitative measures of specific caregiver behaviors, identify which behaviors need to be addressed and/or monitored during infant-caregiver interactions, provide micro-level assessments of which caregiver behaviors are most influenced by experimental manipulation, and analyze longitudinal changes in infant-caregiver interactions. Moreover, the use of sequential data analysis can be extended and applied to any type of interaction. It is a versatile statistical technique that is useful for examining all types of behaviors.

## Figures and Tables

**Table 1:** Descriptions of categories used to classify behaviors.

Behavior	Description
Object-related non-vocal	Non-verbal behaviors that involve an object (e.g., manipulating, showing, pointing at, looking at, or getting a toy)
Object-related vocal	a Infant: Any object-related behavior paired with a vocalization that refers to the object (e.g., infant makes an object-directed vocalization while looking at a ball) b Caregiver: Any object-related behavior paired with a vocalization that refers to the object; must be attempting to reorganize the infant’s attention towards the object (e.g., infant makes an object-related vocalization while looking at a ball; caregiver says, “Look at the ball!”)
Dyadic non-vocal	Face-to-face interaction that involves eye contact and/or physical contact (e.g., touching)
Dyadic vocal	Any dyadic behavior paired with a non-cry vocalization (e.g., babbling, cooing, talking)
Distress vocalization (coded only for infants)	Crying or extreme fussing
Other vocal	Any vocalization that does not fit within the above vocal categories (i.e., object-related vocal, dyadic vocal, distress vocalization)
Other object-related non-vocal (coded only for caregivers)	Any object-related non-vocal behavior that does not involve interaction with the infant (e.g., manipulating an object other than the one the infant is focused on)

**Table 2:** Repeating analysis transitional probabilities: Session 1.^a^

	ion	iov	idn	idv	icry	iot	con	cov	cdn	cdv	cot	coo
ion	**0.356**	0.070	0.003	0.006	0.001	0.007	0.164	0.133	0.033	0.188	0.007	0.032
iov	**0.313**	0.132	0.008	0.002	0.001	0.002	0.122	0.217	0.016	0.123	0.018	0.046
idn	0.079	0.013	0.129	0.040	0.079	0.008	0.092	0.019	**0.287**	0.250	0.000	0.002
idv	0.203	0.015	0.090	0.139	0.000	0.000	0.113	0.030	0.113	**0.289**	0.000	0.008
icry	0.039	0.000	0.140	0.000	0.275	0.000	0.043	0.014	0.159	**0.275**	0.014	0.039
iot	0.129	0.004	0.019	0.002	0.000	**0.294**	0.081	0.027	0.080	0.278	0.011	0.076
con	**0.496**	0.044	0.014	0.007	0.002	0.010	0.207	0.097	0.009	0.109	0.001	0.004
cov	**0.406**	0.066	0.003	0.002	0.001	0.005	0.079	0.381	0.004	0.026	0.005	0.021
cdn	0.253	0.013	0.092	0.022	0.022	0.027	0.026	0.006	0.242	**0.294**	0.001	0.004
cdv	**0.442**	0.028	0.030	0.016	0.011	0.033	0.090	0.028	0.079	0.224	0.002	0.017
cot	0.222	0.060	0.000	0.000	0.009	0.014	0.017	0.103	0.006	0.051	0.211	**0.308**
coo	**0.334**	0.067	0.001	0.001	0.005	0.038	0.023	0.103	0.001	0.083	0.128	0.216

Behavior abbreviations: ion, infant object-related non-vocal; iov, infant object-related vocal; idn, infant dyadic non-vocal; idv, infant dyadic vocal; icry, infant cry; iot, infant other vocal; con, caregiver object-related non-vocal; cov, caregiver object-related vocal; cdn, caregiver dyadic non-vocal; cdv, caregiver dyadic vocal; cot, caregiver other vocal; coo, caregiver other non-vocal.

a Behavior with the largest transitional probability denoted in bold.

**Table 3:** Repeating analysis transitional probabilities: Session 2/3.^a^

	ion	iov	idn	idv	icry	iot	con	cov	cdn	cdv	cot	coo
ion	**0.358**	0.064	0.002	0.004	0.000	0.003	0.159	0.124	0.027	0.243	0.003	0.012
iov	**0.335**	0.102	0.003	0.002	0.001	0.003	0.106	0.223	0.019	0.173	0.008	0.023
idn	0.042	0.005	0.127	0.074	0.005	0.023	0.077	0.040	0.271	**0.334**	0.000	0.000
idv	0.129	0.002	0.073	0.083	0.000	0.006	0.100	0.044	0.226	**0.336**	0.000	0.002
icry	0.000	0.000	0.037	0.000	0.183	0.012	0.024	0.012	0.317	**0.402**	0.000	0.012
iot	0.074	0.007	0.023	0.006	0.001	0.280	0.134	0.023	0.075	**0.331**	0.001	0.044
con	**0.456**	0.038	0.010	0.006	0.000	0.015	0.195	0.108	0.007	0.161	0.001	0.002
cov	**0.389**	0.066	0.005	0.003	0.000	0.003	0.095	0.380	0.004	0.038	0.004	0.012
cdn	0.221	0.019	0.081	0.040	0.010	0.025	0.017	0.012	0.227	**0.346**	0.000	0.002
cdv	**0.463**	0.027	0.027	0.016	0.004	0.021	0.101	0.031	0.068	0.236	0.001	0.007
cot	**0.255**	0.071	0.000	0.000	0.000	0.004	0.008	0.151	0.000	0.054	0.213	0.243
coo	**0.305**	0.078	0.000	0.000	0.000	0.039	0.025	0.094	0.001	0.130	0.112	0.214

Behavior abbreviations: ion, infant object-related non-vocal; iov, infant object-related vocal; idn, infant dyadic non-vocal; idv, infant dyadic vocal; icry, infant cry; iot, infant other vocal; con, caregiver object-related non-vocal; cov, caregiver object-related vocal; cdn, caregiver dyadic non-vocal; cdv, caregiver dyadic vocal; cot, caregiver other vocal; coo, caregiver other non-vocal.

a Behavior with the largest transitional probability denoted in bold.

**Table 4:** Non-repeating analysis transitional probabilities: Session 1.^a^

	ion	iov	idn	idv	icry	iot	con	cov	cdn	cdv	cot	coo
ion	–	0.108	0.005	0.009	0.001	0.011	0.255	0.207	0.051	**0.292**	0.011	0.050
iov	**0.361**	–	0.009	0.002	0.001	0.002	0.141	0.250	0.018	0.142	0.021	0.054
idn	0.091	0.015	–	0.046	0.091	0.009	0.106	0.022	**0.330**	0.288	0.000	0.002
idv	0.236	0.017	0.105	–	0.000	0.000	0.131	0.035	0.131	**0.336**	0.000	0.009
icry	0.053	0.000	0.193	0.000	–	0.000	0.060	0.020	0.220	**0.380**	0.020	0.053
iot	0.182	0.005	0.027	0.003	0.000	–	0.115	0.038	0.113	**0.394**	0.016	0.107
con	**0.626**	0.056	0.018	0.008	0.002	0.013	–	0.122	0.011	0.138	0.001	0.004
cov	**0.656**	0.107	0.005	0.004	0.002	0.008	0.127	–	0.006	0.042	0.008	0.035
cdn	0.334	0.017	0.122	0.028	0.029	0.035	0.034	0.008	–	**0.388**	0.001	0.005
cdv	**0.570**	0.036	0.039	0.020	0.014	0.042	0.116	0.036	0.102	–	0.003	0.022
cot	0.282	0.076	0.000	0.000	0.011	0.018	0.022	0.130	0.007	0.065	–	**0.390**
coo	**0.426**	0.086	0.001	0.001	0.007	0.048	0.030	0.132	0.001	0.105	0.163	–

Behavior abbreviations: ion, infant object-related non-vocal; iov, infant object-related vocal; idn, infant dyadic non-vocal; idv, infant dyadic vocal; icry, infant cry; iot, infant other vocal; con, caregiver object-related non-vocal; cov, caregiver object-related vocal; cdn, caregiver dyadic non-vocal; cdv, caregiver dyadic vocal; cot, caregiver other vocal; coo, caregiver other non-vocal.

a Behavior with the largest transitional probability denoted in bold.

**Table 5:** Non-repeating analysis transitional probabilities: Session 2/3.^a^

	ion	iov	idn	idv	icry	iot	con	cov	cdn	cdv	cot	coo
ion	–	0.100	0.004	0.007	0.000	0.005	0.248	0.193	0.042	**0.378**	0.005	0.018
iov	**0.373**	–	0.004	0.003	0.001	0.004	0.118	0.248	0.022	0.193	0.009	0.026
idn	0.049	0.006	–	0.085	0.006	0.027	0.088	0.046	0.311	**0.383**	0.000	0.000
idv	0.140	0.002	0.079	–	0.000	0.007	0.109	0.048	0.247	**0.367**	0.000	0.002
icry	0.000	0.000	0.045	0.000	–	0.015	0.030	0.015	0.388	**0.493**	0.000	0.015
iot	0.103	0.010	0.032	0.008	0.002	–	0.185	0.032	0.105	**0.460**	0.002	0.060
con	**0.567**	0.047	0.012	0.008	0.000	0.019	–	0.135	0.009	0.200	0.001	0.003
cov	**0.627**	0.107	0.009	0.005	0.000	0.006	0.153	–	0.006	0.062	0.006	0.020
cdn	0.286	0.025	0.105	0.051	0.013	0.033	0.022	0.016	–	**0.447**	0.000	0.002
cdv	**0.605**	0.035	0.035	0.020	0.005	0.028	0.132	0.040	0.089	–	0.001	0.009
cot	**0.324**	0.090	0.000	0.000	0.000	0.005	0.011	0.191	0.000	0.069	–	0.309
coo	**0.389**	0.099	0.000	0.000	0.000	0.050	0.032	0.120	0.002	0.166	0.143	–

Behavior abbreviations: ion, infant object-related non-vocal; iov, infant object-related vocal; idn, infant dyadic non-vocal; idv, infant dyadic vocal; icry, infant cry; iot, infant other vocal; con, caregiver object-related non-vocal; cov, caregiver object-related vocal; cdn, caregiver dyadic non-vocal; cdv, caregiver dyadic vocal; cot, caregiver other vocal; coo, caregiver other non-vocal.

a Behavior with the largest transitional probability denoted in bold.

## References

[1] Hart B., Risley T.R. (1995). Meaningful Differences in the Everyday Experiences of Young American Children.

[2] Hoff E. (2003). The specificity of environmental influence: socioeconomic status affects early vocabulary development via maternal speech. Child Dev..

[3] Bradley R.H., Caldwell B.M. (1984). The relation of infants’ home environments to achievement test performance in first grade: a follow-up study. Child Dev..

[4] Bradley R.H., Caldwell B.M., Rock S.L. (1988). Home environment and school performance: a ten-year follow-up and examination of three models of environmental action. Child Dev..

[5] Fernald A., Marchman V.A., Weisleder A. (2013). SES differences in language processing skill and vocabulary are evident at 18 months. Dev. Sci..

[6] Walker D., Greenwood C., Hart B., Carta J. (1994). Prediction of school outcomes based on early language production and socioeconomic factors. Child Dev..

[7] Barbosa P.G., Carodoso-Martins C., Echols C. (2016). Child-directed speech and its impact on early vocabulary acquisition: evidence from Brazilian Portuguese. Psychol. Neurosci..

[8] Riksen-Walraven J.M. (1978). Effects of caregiver behavior on habituation rate and self-efficacy in infants. Int. J. Behav. Dev..

[9] Tamis-LeMonda C.S., Bornstein M.H. (2002). Maternal responsiveness and early language acquisition. Adv. Child Dev. Behav..

[10] Bobbitt R.A., Gourevitch V.P., Miller L.E., Jensen G.D. (1969). Dynamics of social interactive behavior: a computerized procedure for analyzing trends, patterns, and sequences. Psychol. Bull..

[11] Jeong A. (2005). A guide to analyzing message-response sequences and group interaction patterns in computer-mediated communication. Distance Educ..

[12] Kogan K.L., Wimberger H.C. (1966). An approach to defining mother-child interaction styles. Percept. Mot. Skills.

[13] Lii S.Y. (1981). The group comparison of different sequential behavioral analyses on dyadic interaction. J. Exp. Educ..

[14] Sigel I.E., Parke R.D. (1987). Structural analysis of parent-child research models. J. Appl. Dev. Psychol..

[15] Gros-Louis J.G., West M.J., Goldstein M.H., King A.P. (2006). Mothers provide differential feedback to infants’ prelinguistic sounds. Int. J. Behav. Dev..

[16] Bornstein M.H., Tamis-LeMonda C.S., Tal J., Ludemann P., Toda S., Rahn C., Pêcheux M., Azuma H., Vardi D. (1992). Maternal responsiveness to infants in three societies: the United States, France, and Japan. Child Dev..

[17] Vollmer L. (2007). Developmental Changes in Infants’ Knowledge of the Instrumental Value of Babbling.

[18] Bakeman R., Quera V. (1995). Log-linear approaches to lag-sequential analysis when consecutive codes may and cannot repeat. Psychol. Bull..

[19] O’Connor B.P. (1999). Simple and flexible SAS and SPSS programs for analyzing lag-sequential categorical data. Behav. Res. Methods Instrum. Comput..

